# Primary tubercular osteomyelitis of lateral orbital wall: a rare presentation

**DOI:** 10.1186/s12348-025-00518-1

**Published:** 2025-12-11

**Authors:** Vandana Sharma, Vikasdeep Gupta, Harmeet Kaur, Anuradha Raj

**Affiliations:** 1 Department of Ophthalmology, AIIMS, Jodhpur Romana, Room no 2213, Dabwali Road, Bathinda, India; 2 AIIMS, Bathinda, India

## Abstract

Osteomyelitis of facial bones is a very rare presentation. Primary presentation of tubercular osteomyelitis in facial bones is even rarer. We are reporting a case of zygomatic tubercular osteomyelitis presenting in a very innocuous way. Identification of such cases in early stage can help in timely intervention. A 30 years old female presented to Ophthalmology Out Patient Department (OPD) with complain of a mildly painful nodule over left lower eyelid, turning into an ulcer. After appropriate investigation, diagnosis of primary tubercular osteomyelitis of left zygomatic bone was made and patient underwent debridement and anti-tubercular treatment. Patient has healed satisfactorily, with no sinus formation, no residual bony on filtration and good cosmetic results. Early diagnosis and timely management of such cases can avoid extensive involvement of bone, thereby reducing the subsequent morbidity of the patients.

Tubercular osteomyelitis commonly involves long bones and the spine. However, rarely, it may also involve the midfacial bones. Out of these, involvement of lateral orbital wall with the disease as a primary presentation of tuberculosis is even rarer [1]. Predisposing factors for skull involvement are malnutrition, poor socioeconomic status, and immunosuppression [2]. Frontal and parietal bones are most commonly involved [3].

## Case presentation

A 30 year old female presented to Ophthalmology OPD with a complaint of mildly painful ulcer over left lower eyelid for the last three days. The ulcer was preceded by a painful non erythematous nodular lesion over the region noticed 15 days earlier which burst spontaneously. Patient did not have any other systemic or ocular complaints.

**Fig. 1:**
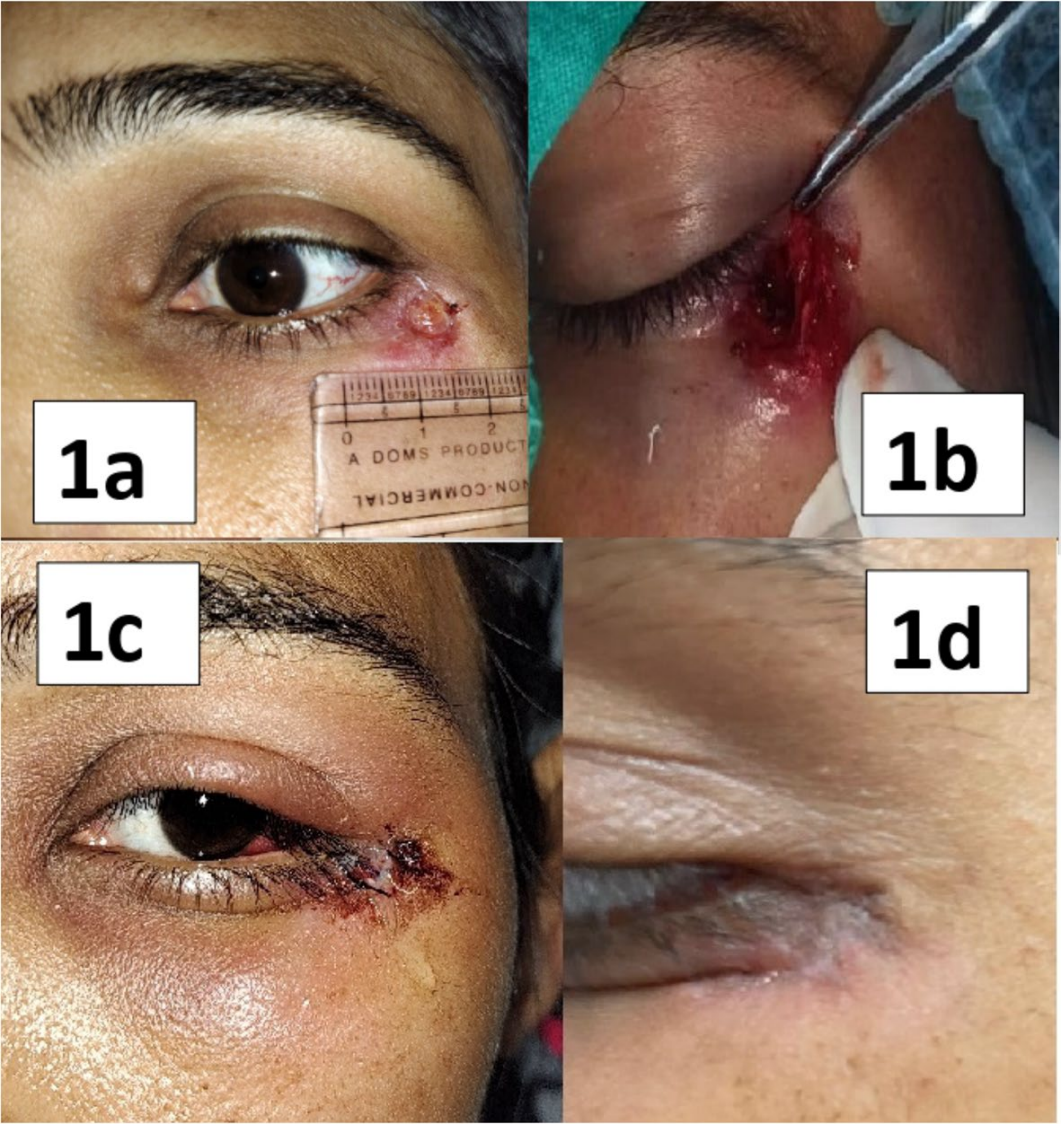
**a** ulcer with undermined, no erythematous edges measuring 8 mm horizontally and vertically with minimal purulent discharge over floor of ulcer. **b**: intraoperative photograph showing debridement of involved bone; **c** and **d**: appearance of wound postoperatively on day 3 (**c**) and on day 7 (**d**) respectively

On examination, the ulcer was circular with a diameter of about 6 mm located at the junction of lateral wall and floor of orbit (Fig. 1a). The skin around the ulcer was adherent to underlying bone and was non tender. No collection was palpable under the lesion and minimal purulent discharge was visible on the floor of the lesion. No preauricular, submental, submandibular or cervical lymphadenopathy was present. Patient was started on oral antibiotics empirically (amoxycillin with potassium clavulanate 1.2 gm three times a day with metrinodazole 400 mg three times a day) as a preliminary diagnosis of chronic osteomyelitis of zygoma with discharging sinus was made. Patient was given standard antibiotic therapy for two weeks. However no improvement was noted.

**Fig. 2:**
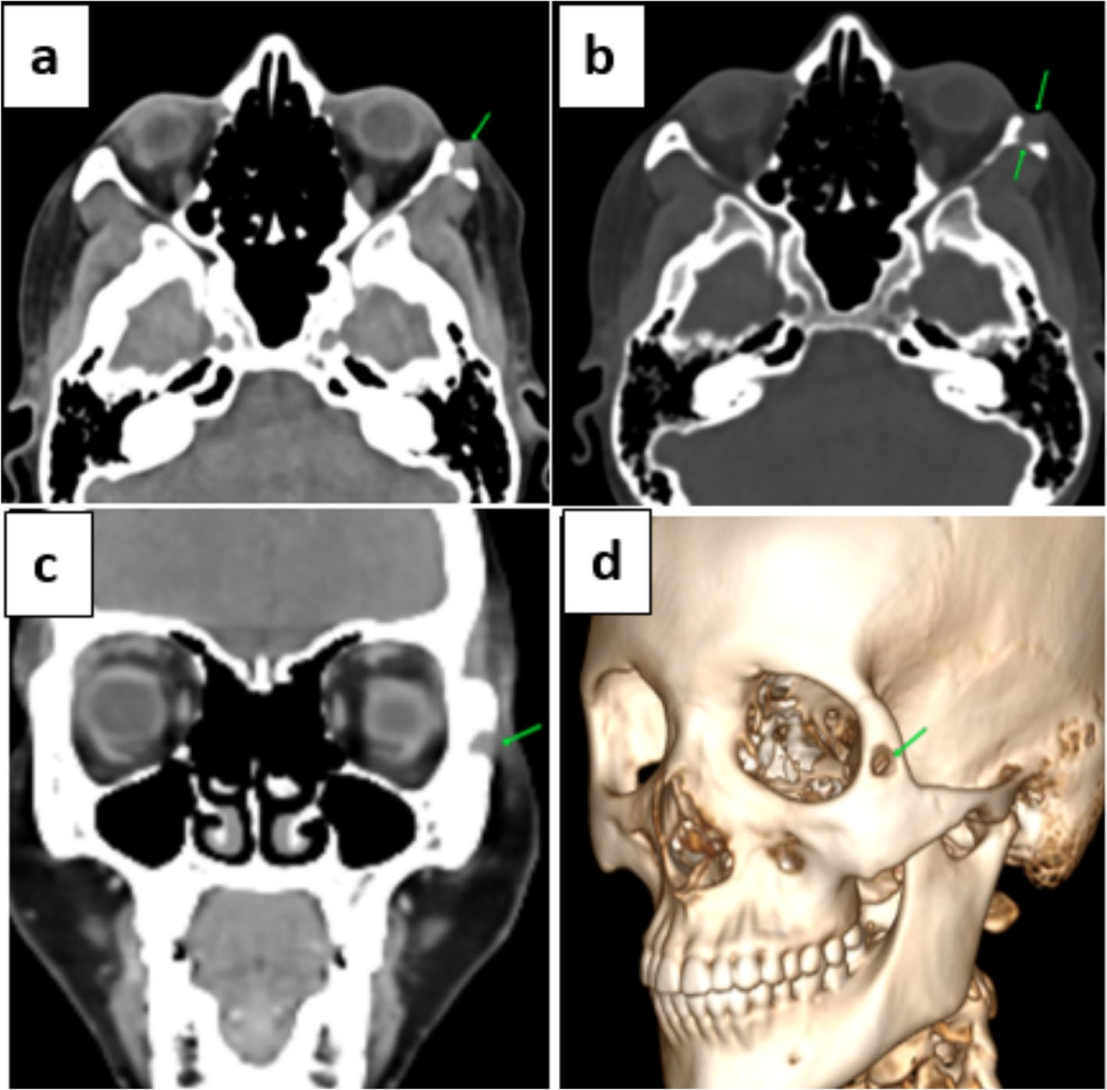
Left zygoma Osteomyelitis. **a** axial NCCT (soft tissue window), **b** axial NCCT (bone window), (**c**) coronal CECT (soft tissue window) & (**d**) oblique sagittal VRT images of the face show an ill-defined osteolytic lesion involving left zygomatic bone with associated sinus tract and mildly enhancing soft tissue thickening (green arrows). NCCT: Non-contrast Computed tomography, CECT: Contrast-enhanced Computed tomography, VRT: Volume Rendering Technique

Contrast enhanced Computed Tomography of the face revealed an ill-defined osteolytic lesion of zygomatic bone on left side with associated sinus tract and mildly enhancing soft tissue thickening (Fig. 2) and no deep extension of the pathology into infratemporal fossa.

Debridement of the diseased bone was done by the ENT surgeon (Fig. 1b, c and d). Blood investigations revealed a elevated erythrocyte sedimentation rate. Patient did not undergo a preoperative Mantoux or IGRA test. Histopathological examination found well-formed epithelioid cell granulomas with central caseating necrosis and multinucleated Langhans’ Giant cells. Ziehl Neelsen stain for acid fast bacilli was positive. Diagnosis was revised to tubercular (TB) osteomyelitis of the zygomatic bone. Chest radiograph did not reveal any primary focus. No there was no enlargement of any lymph nodes in any other part of the body as assessed by palpation of axillary nodes and ultrasonography of abdominal nodes. Additionally, the patient did not have any constitutional symptoms suggestive of tuberculosis. Patient was not immunocompromised and did not elicit any prior history of contact with a case of tuberculosis. Cartridge Based Nucleic Acid Amplification Test (CBNAAT) performed on the debrided tissue was positive. Therefore, the involvement can be considered to be a primary presentation of tuberculosis in this patient.

Patient was started on anti-tubercular treatment as per NTEP [4] guidelines, wherein the intensive phase consists of 8 weeks of isoniazid (5 mg/kg daily (4 to 6 mg/kg)), rifampicin (10 mg/kg daily (8 to 12 mg/kg)), pyrazinamide (25 mg/kg daily (20 to 30 mg/kg)) and ethambutol (15 mg/kg daily (12 to 18 mg/kg)) given under direct observation in daily dosages as per weight band categories. Continuation phase consists of 16 weeks of isoniazid, rifampicin and ethambutol in daily dosages. The continuation phase may be extended upto 24 weeks in Skeletal TB. However, in this case the lesion was small and radiologically healed after the initial 16 weeks of continuation phase. Therefore no extension was needed in this case. The wound has healed without any sinus formation Isoniazid (H) Rifampicin (R) Pyrazinamide (Z) Ethambutol (E) Streptomycin (S)* Doses 15 mg/kg daily (15 to 20 mg/kg).

## Discussion

Orbital TB has been clinically classified into five categories: classical periostitis, orbital tuberculoma with no bony destruction, orbital TB with evidence of bony destruction (not classified as classical periostitis), orbital TB as a result of spread from paranasal sinuses and dacryoadenitis [5]. This patient suffered from orbital TB with evidence of bony destruction radiologically as well as surgically.

Therefore we should keep a high index of suspicion in such cases with atypical presentation especially in endemic areas. Diagnosing tuberculosis in such cases can be challenging as it may be difficult to identify the mycobacteria due to their small number. A new modality of investigation i.e., CBNAAT can be of help in identifying the small number of mycobacteria in tissue as well as fluids. While it a fast and accurate test with results comparable with culture; high cost, need for a stable electricity supply, replenishment of the cartridges every 18 months, and a stable temperature ceiling limit its application to all cases [6].

Treatment essentially constitutes use of anti-tubercular drugs as per the NTEP guidelines [4]. Patient must be motivated to be compliant with the drug regimen to avoid development of drug resistance and complete cure. Surgical debridement can be done in extensive disease but there are chances of sinus formation which are difficult to get rid of later on. Debridement was done in this case as a preliminary microbiological examination of the pus discharge did not reveal any Acid Fast Bacilli.

## Data Availability

No datasets were generated or analysed during the current study.
